# Development of fully automated deep-learning-based approach for prediction of sentinel lymph node metastasis in breast cancer patients using ultrasound imaging

**DOI:** 10.3389/fonc.2025.1592521

**Published:** 2025-08-28

**Authors:** Taixia Liu, Guojia Zhao, Wei Wei, Qingling Zhang, Jing Wu, Xuying Chen, Dong Liu, Xiangming Zhu

**Affiliations:** ^1^ Cheeloo College of Medicine, Shandong University, Jinan, Shandong, China; ^2^ Department of Ultrasound, The First Affiliated Hospital of University of Science and Technology of China (USTC), Division of Life Sciences and Medicine, University of Science and Technology of China, Hefei, Anhui, China; ^3^ Department of Ultrasound, Shanghai Municipal Hospital of Traditional Chinese Medicine, Shanghai, China; ^4^ Department of Ultrasound, Lin Yi People’s Hospital, Linyi, Shandong, China; ^5^ Department of Ultrasound, The First Affiliated Hospital of Wannan Medical College, Wuhu, Anhui, China; ^6^ Department of Information, The First Affiliated Hospital of Wannan Medical College, Wuhu, Anhui, China

**Keywords:** automatic segmentation, deep learning, prediction model, sentinel lymph node metastasis, breast cancer

## Abstract

**Purpose:**

This study aimed to develop a novel predicting model based on deep learning (DL) to predict sentinel lymph node (SLN) metastasis in breast cancer (BC) patients using ultrasound (US) imaging.

**Methods:**

A retrospective cohort consisting of 692 female BC patients from two hospitals was analyzed, with data collected from January 2020 to October 2023. Patients from Hospital A were randomly allocated to training (*n* = 405) and internal validation (*n* = 174) sets (7:3 ratio), with Hospital B patients (*n* = 113) serving as the external test set. A post-fusion model integrating the DeepLabV3, U-Net, and U-Net++ segmentation algorithms, respectively, was utilized to automatically delineate regions of interest (ROIs). Furthermore, three convolutional neural networks (CNNs)—ResNet50, ResNet101, and DenseNet121, respectively—were employed to analyze the cropped regions and concurrently construct a predictive model. A composite model that incorporates the DL signature (DL Sig) alongside clinical factors was developed by utilizing logistic regression (LR). A database to compare human and machine performance was created to evaluate the model’s effectiveness. A nomogram was ultimately constructed to forecast the occurrence of SLN metastasis. The evaluation of model performance involved the utilization of receiver operating characteristic (ROC) curves, calibration curves, and decision curve analysis (DCA), respectively.

**Results:**

The post-fusion model demonstrated a robust correlation with manual delineation, yielding Dice coefficients of 0.893 and 0.855 in the internal validation and external test sets, respectively. The ResNet50 model, recognized as the most effective base model, demonstrated an area under the curve (AUC) of 0.773 (95% CI: 0.706–0.840) and an accuracy of 68% in the internal validation set (VS). In the external test set (TS), it achieved 0.765 AUC (95% CI: 0.674–0.856) with accuracy of 74%. The integrated model, which combined the DL Sig with clinical factors, exhibited the most effective performance in forecasting SLN metastasis, achieving 0.763 AUC (95% CI: 0.671–0.855) with accuracy of 69% in the TS. The DCA demonstrated notable clinical utility in the integrated model, surpassing the performance of both senior and junior radiologists.

**Conclusion:**

Our novel predictive model exhibited superior performance compared to both senior and junior radiologists in predicting SLN metastasis. Its capability for automatic segmentation and prediction highlights its potential for clinical applications.

## Introduction

Breast cancer (BC) ranks as a prevalent malignancy among women globally and stands as the foremost cause of cancer incidence worldwide (1, 2). Axillary lymph node (ALN) metastasis crucially affects prognostic evaluations, clinical staging, and the refinement of therapeutic strategies for BC patients (3, 4). The status of the sentinel lymph node (SLN) as initial metastatic gateway in BC lymphatic spread could provide critical prognostic information that influences therapeutic strategies (5). Currently, SLN biopsy (SLNB) has increasingly supplanted ALN dissection (ALND) for staging ALNs in node-negative BC patients, primarily due to its less invasive nature (6). However, a substantial percentage of patients undergoing SLNB demonstrate negative SLN results, implying that unnecessary SLNB may lead to overtreatment (7, 8). Additionally, despite its less invasiveness, SLNB carries potential risks and complications (9). To address the limitations of SLNB, it is imperative to establish a non-invasive and efficacious approach for predicting SLN metastasis.

Ultrasound (US), mammography, computed tomography (CT) scans, and magnetic resonance imaging (MRI) serve as the principal non-invasive imaging modalities for predicting SLN metastasis. US serves as the preferred initial assessment method for the prediction of SLN status by evaluating both the intratumoral and peritumoral regions of BC, with achieved moderate AUCs from 0.73 to 0.835 (10–13). However, the diagnostic accuracy remains suboptimal, primarily attributable to the limitations in ultrasonography, such as its inability to provide functional features on breast tumors and its reliance on morphological characteristics (14). Therefore, developing a novel tool is imperative to enhance the precision of US evaluations for SLN status in BC patients while providing quantifiable and clinically interpretable predictive metrics.

In recent years, DL has attracted considerable attention within the medical imaging sector for its robust capabilities in processing large datasets and images (15–17). Convolutional neural networks (CNNs), a foundational architecture in DL, have demonstrated exceptional proficiency in hierarchical feature extraction from medical imaging data (18). Prior research (19) showed that deep-learning-derived features extracted from US images achieved promising accuracy in predicting SLN metastasis in BC, with an AUC of 0.85 (training set). However, some existing studies (20, 21) that rely on manual or semiautomated techniques have been recognized as often being laborious and time-consuming, which can compromise the consistency and reproducibility of the results. Therefore, automatic segmentation is crucial to achieve more accurate results in the era of precision medicine.

Image segmentation plays a pivotal role in analysis, incorporating detection, feature extraction, classification, and treatment (22, 23). Furthermore, many recent studies (24–27) have delved into segmentation techniques for breast tumors in US images, yet challenges persist owing to significant speckle noise and the diverse morphology of tumors in US images. Additionally, contemporary segmentation algorithms like DeepLabV3 (28) and U-Net (29) require further advancements to enhance semantic segmentation capabilities. In this study, we introduce an automated DL model that harmoniously combines DeepLabV3, U-Net, and U-Net++ for improved feature extraction and segmentation accuracy. Subsequently, we validated an integrated model based on DL in US images. Finally, we employed a nomogram, which holds promise as a quantifiable, interpretable, and clinically accessible tool for determining SLN status in BC patients.

## Patients and methods

### Patient population

**Figure 1:**
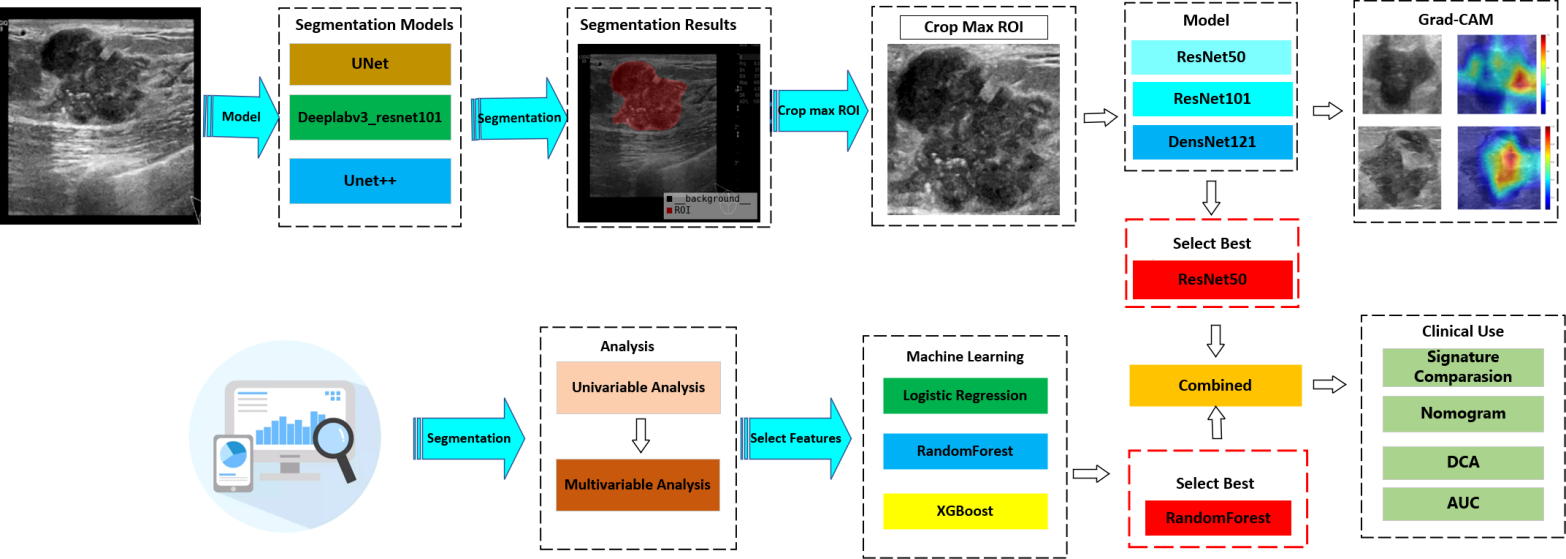
Workflow of this study.

**Figure 2:**
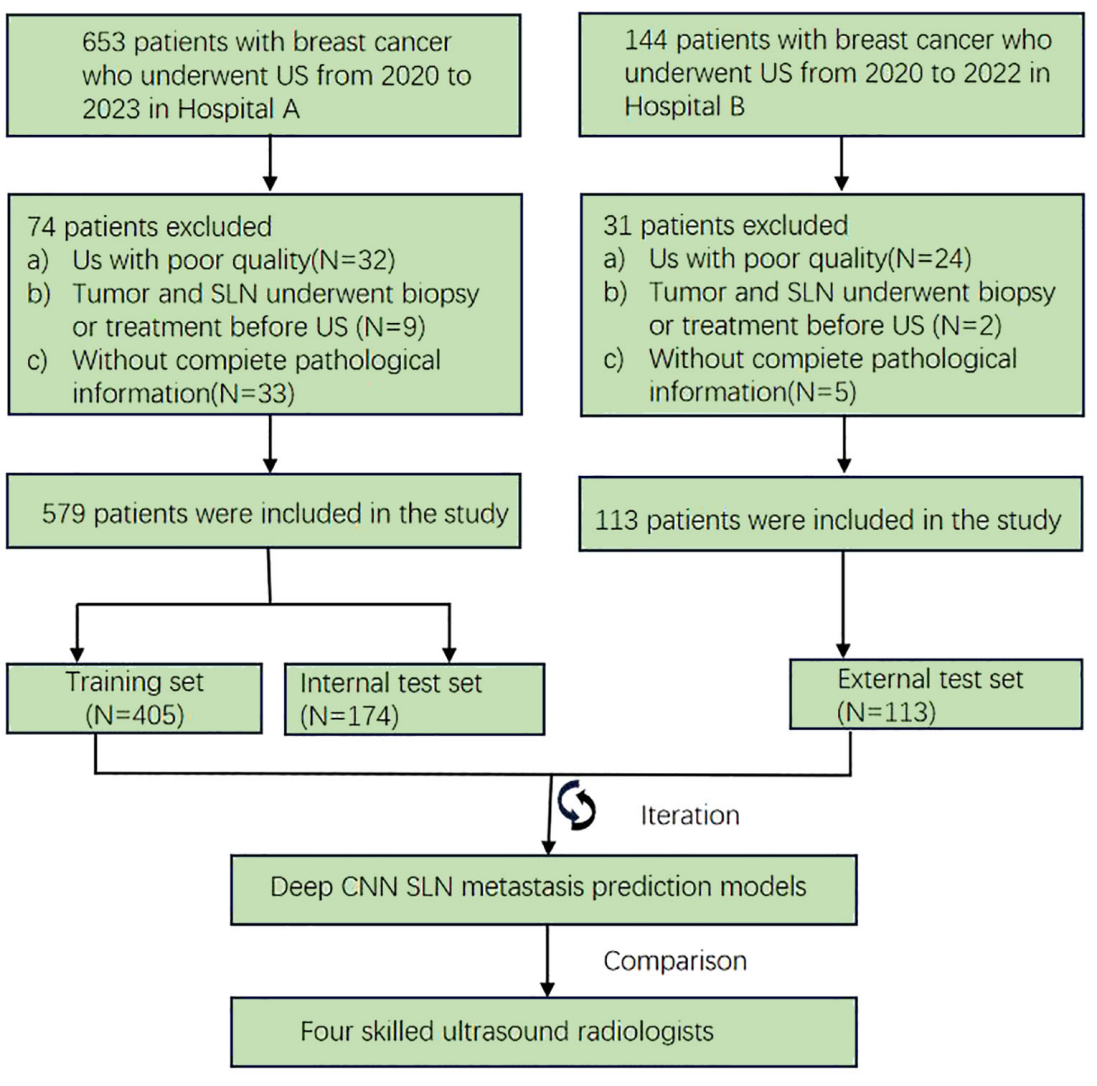
Dataset configuration of this study.

From January 2020 to December 2023, a retrospective study was conducted on 692 BC patients from two hospitals—The Linyi People’s Hospital (A) and the First Affiliated Hospital of Wannan Medical College (B). Patients from Hospital A were randomly allocated to training (*n* = 405) and internal validation (*n* = 174) sets (7:3 ratio), with Hospital B patients (*n* = 113) serving as the external test set. The study protocol adhered strictly to the ethical standards of the Declaration of Helsinki (2013). The ethics committee approved this retrospective study and waived the need for informed consent. Patients who underwent US-guided needle biopsy or surgery within the week of diagnosis were chosen. Further detailed recruitment criteria include (I) histologically confirmed malignant breast tumors, (II) known SLN metastasis status from final histopathology, (III) known molecular subtypes, and (IV) surgical or puncture pathological results for breast lesions. The exclusion criteria included (I) incomplete clinical data, immunohistochemistry, or pathology results, (II) male BC patients, (III) prior biopsy intervention, chemotherapy, or radiofrequency ablation, and (IV) poor image quality or absence of SLNB or ALND. The workflow for constructing the model is depicted in **Figure 1**, while the flow chart for recruiting new patients is illustrated in **Figure 2**.

### Data sets

Baseline clinical and histopathological data, including age, lesion sizes, histological grade, histological type, immunohistochemistry (IHC) results, and the SLN status, were obtained from the patient’s medical records as detailed in **Table 1**. The status of estrogen receptors (ER), progesterone receptors (PR), human epidermal growth factor receptor 2 (HER2), and Ki-67, respectively, were used to evaluate by *in situ* hybridization (IHC) and fluorescence *in situ* hybridization (FISH). The diagnosis of breast lesions relied on pathology reports, which were considered the gold standard.

**Table 1:** Baseline characteristics of population.

Characteristic	Training set (n=405)		pvalue	Validation set (n = 174)		pvalue	Test set (n=113)		pvalue
	SLN-(n = 216)	SLN+(n = 189)		SLN-(n = 88)	SLN+(n = 86)		SLN-(n = 57)	SLN+(n = 56)	
Age (mean ± SD), years	50.93±10.28	51.57±9.92	0.522	49.58±9.49	50.88±11.58	0.417	52.44±11.35	52.52±9.58	0.968
Size (mean ± SD), cm	22.90±12.32	24.01±11.01	0.099	20.06±8.56	24.01±10.49	0.005*	21.60±7.97	24.88±9.48	0.062
Histologic_type (%)			<0.001*			<0.001*			0.110
Invasive ductal	161(74.54)	182(96.30)		63(71.59)	81(94.19)		48(84.21)	46(82.14)	
Invasive lobular	2(0.93)	4(2.12)		2(2.27)	3(3.49)		null	null	
DCIS	44(20.37)	1(0.53)		21(23.86)	1(1.16)		2(3.51)	7(12.50)	
Other pathology types	9(4.17)	2(1.06)		2(2.27)	1(1.16)		7(12.28)	3(5.36)	
Histologic_grade (%)			0.029*			0.437			0.978
I	14(6.48)	5(2.65)		6(6.82)	4(4.65)		9(15.79)	8(14.29)	
II	153(70.83)	123(65.08)		55(62.50)	48(55.81)		35(61.40)	37(60.07)	
III	49(22.69)	61(32.28)		27(30.68)	34(39.53)		13(22.81)	11(19.64)	
ER status (%)			0.063			0.920			0.916
Negative	63(29.17)	39(20.63)		26(29.55)	27(31.40)		26(45.61)	24(42.86)	
Positive	153(70.83)	150(79.37)		62(70.45)	59(68.60)		31(54.39)	32(57.14)	
PR status (%)			0.027*			0.668			0.910
Negative	78(36.11)	48(25.40)		29(32.95)	32(37.21)		33(57.89)	34(60.71)	
Positive	138(63.89)	141(74.60)		59(67.05)	54(62.79)		24(42.11)	22(39.29)	
Her_2 status (%)			0.119			0.282			0.595
Negative	155(71.76)	121(64.02)		63(71.59)	54(62.79)		42(73.68)	41(73.21)	
Positive	61(28.24)	68(35.98)		25(28.41)	32(37.21)		15(26.32)	15(26.79)	
Ki_67 status (%)			0.413			0.424			0.441
Negative	92(42.59)	72(38.10)		39(44.32)	32(37.21)		10(17.54)	6(10.71)	
Positive	124(57.41)	117(61.90)		49(55.68)	54(62.79)		47(82.46)	50(89.29)	

SLN-, sentinel lymph node negative; SLN+, sentinel lymph node positive; SD, standard deviation; DCIS, ductal carcinoma in situ; ER, estrogen receptor; PR, progesterone receptor; HER-2, human epidermal growth factor receptor 2. *p < 0.05.

### US image acquisition

The equipment used to collect images in the US came from a variety of manufacturers, including GE LOGIQ E9 (ML6–15 MHz), Siemens Acuson S2000 (4–9 MHz), Esaote Mylab Twice (4–13 MHz), and Philips EPIQ5 (5–12 MHz) transducers. The top chest region was completely exposed as patients were positioned either supine or laterally. At the center was the lesion, which formed the focal zone. The images were captured by an experienced sonographer specializing in breast imaging for over 5 years and deposited in the Picture Archiving and Communication System (PACS).

### ROI segmentation

In this study, an automated segmentation algorithm was used to identify ROIs. Additionally, to enhance the accuracy of ROI segmentation, a post-fusion algorithm was introduced to merge various algorithms.

#### Images for segmentation and preprocessing

Manual ROI was conducted using ITK-SNAP (version 3.8.0), with careful delineation of tumor boundaries performed on the largest cross-sectional slice of each lesion. The tumor’s lobules and burrs, as well as the lesion’s perimeter, were used to define the ROIs. Both radiologist A and radiologist B (8 and 12 years of breast US practice, respectively) independently conducted ROI delineation on a randomly selected sample of 50 patients. Reliability (both inter- and intra-rater) was quantified via Intraclass Correlation Coefficient (ICC) analysis, adopting the threshold of ICC ≥0.75 for substantial consistency, while the Dice similarity coefficient (DSC) was employed to quantify spatial overlap agreement between segmentations, with a DSC value ≥0.84 considered indicative of good concordance. The details are illustrated in **Appendix 1**. These initial manual segmentations served as the ground truth dataset for constructing the automated segmentation model. The intensity distribution across RGB channels was standardized by Z-score normalization of the US pictures to minimize variations caused by parameter inconsistency between different machines and imaging modes. Our model was then fed these standardized inputs.

#### Segmentation model training and postprocessing

We comprehensively evaluated the contemporary image segmentation algorithms, including DeepLabV3, U-Net, and U-Net++. All architectures were fine-tuned using transfer learning approaches, with initial parameter optimization performed on the Microsoft Common Objects in Context (MS COCO) dataset. U-Net is noted for its simplicity and effectiveness with limited data, and U-Net++ enhances accuracy with nested skip connections, while DeepLabV3 is recognized for its excellence in semantic segmentation tasks by capturing multi-scale contextual information. To synthesize the outcomes of these algorithms, a post-fusion model for segmentation was proposed. The detailed workflow is illustrated in **Supplementary Figure 1**.

Random image patches were extracted and labeled as positive or negative samples. The numbers of samples and their sizes were defined. Online data augmentation methods, including random cropping and spatial transformations, were implemented to enhance the training dataset diversity. For this task, we turned to the DiceCELoss function, which merges the Dice loss and cross-entropy loss techniques. When the Adam optimizer was first implemented, a learning rate of 1e-3 was utilized. The training protocol consisted of 18,000 iterative updates (600 epochs), using 32 early halting rounds. Throughout the training procedure, an NVIDIA 4090 GPU was utilized, which was running MONAI 0.8.1 and PyTorch 1.8.1.

#### Evaluating the performance of the segmentation mode

For the segmentation process, the ROI was compared with radiologist ground truth annotations. The Dice was used to assess the segmentation process by quantitatively assessing spatial overlap between segmentation results and manual ground truth annotations. The evaluation metric of our segmentation model was Dice, precision, recall, as well as the intersection over union (IoU).

### Combined model construction

Despite the fact that manually delineating ROIs during data annotation could have enhanced diagnostic precision, it was avoided to enable full automation of the diagnostic procedure. Rather, all samples’ ROIs were automatically delineated using the automated segmentation model and then used in the following modeling processes.

#### DL model training

This investigation implemented transfer learning using pre-trained ResNet50, ResNet101, and DenseNet121 models as the fundamental frameworks for extracting DL features. These models had undergone initializing with pretrained weights from the ImageNet database. We selected the slice with the largest ROIs for each patient as the representative image. To reduce background noise, we retained solely the smallest rectangular boundary containing the ROIs. Gray values were scaled to a range of 0 to 1 using min–max normalization. Furthermore, all cropped subregions were resampled to a uniform size of 224 × 224 pixels through nearest-neighbor interpolation to preserve discrete pixel values.

To improve model generalizability, we implemented a cosine decay learning rate strategy, which is characterized as follows:


$$\eta_{t}=\eta_{min}^{i}+\frac{1}{2}\left(\eta_{max}^{i}-\eta_{min}^{i}\right)\left(1+\text{cos}\left(\frac{T_{cur}}{T_{i}}\pi \right)\right)$$

where $\eta_{min}^{i}=0$ represents the minimum learning rate, $\eta_{max}^{i}=0.01$ denotes the maximum learning rate, and $T_{i}=90$ defines the total number of training epochs for the iterative optimization procedure, respectively. The model was optimized using stochastic gradient descent (SGD), with softmax cross-entropy serving as the objective function for loss computation.

#### DL signature building

We compared the AUCs of all DL models and selected the one with the best performance. The predicted probabilities generated by the best model were used as the DL Sig.

#### Combined model building

Clinical predictors were identified through both univariate and multivariate analyses. Subsequently, various machine learning models (RandomForest, XGBoost, LR) were employed to construct the clinical signature. To enhance clinical applicability, the clinical signature was integrated with predictions from the DL model using a LR linear model, leading to the development of the integrated model.

### Radiologists’ prediction

A comparative analysis was conducted between the integrated model and experienced radiologists. Four radiologists of varying seniority (junior radiologists 1 and 2 with 4 and 5 years of post-training experience and senior radiologists 3 and 4 with 8 and 12 years of post-training experience, respectively) independently evaluated the US images for all enrolled patients. They were unable to perceive the pathological information. Model performance was evaluated through ROC analysis, with AUC values and 95% confidence intervals (CIs) computed. Comparative analyses of the AUCs were conducted by utilizing the methodology established by DeLong et al.

### Evaluation of the combined model

Histologic type and Her-2 serve as the clinical factors. These elements were used for both multivariate and univariate analyses with DL Sig. Multivariable analyses with backward stepwise elimination (AIC minimization) was employed to identify independent risk factors. Using the variance inflation factor, we checked if the regression model had any multi-collinearity. We constructed a nomogram using the selected variables. The predictive capabilities of the integrated model and fundamental DL models for SLN status were assessed through ROC curve analysis and AUC quantification in the training, validation, and test sets. Negative predictive value (NPV), positive predictive value (PPV), sensitivity, accuracy, and specificity were among the measures computed. We also ran DCA and calibration curves to evaluate the integrated model’s clinical utility and calibration. The Hosmer–Lemeshow test was used to assess the model’s calibration; this test necessitates a Hosmer–Lemeshow statistic of ≥0.05.

### Statistical analysis

Data were analyzed by utilizing SPSS (version 25.0, IBM), Python (version 3.7.12), and R software (version 3.3.4). Continuous variables were summarized using mean ± standard deviation (SD) for normally distributed data, while categorical variables were presented as absolute frequencies and percentages (*n*, %). Categorical variables were analyzed using Pearson’s chi-square test or Fisher’s exact test, Continuous variables were compared using Mann–Whitney *U*-test. The variations in AUC values were evaluated through the DeLong test, with *p <*0.05 deemed statistically significant.

## Results

### Baseline characteristics

**Figure 3:**
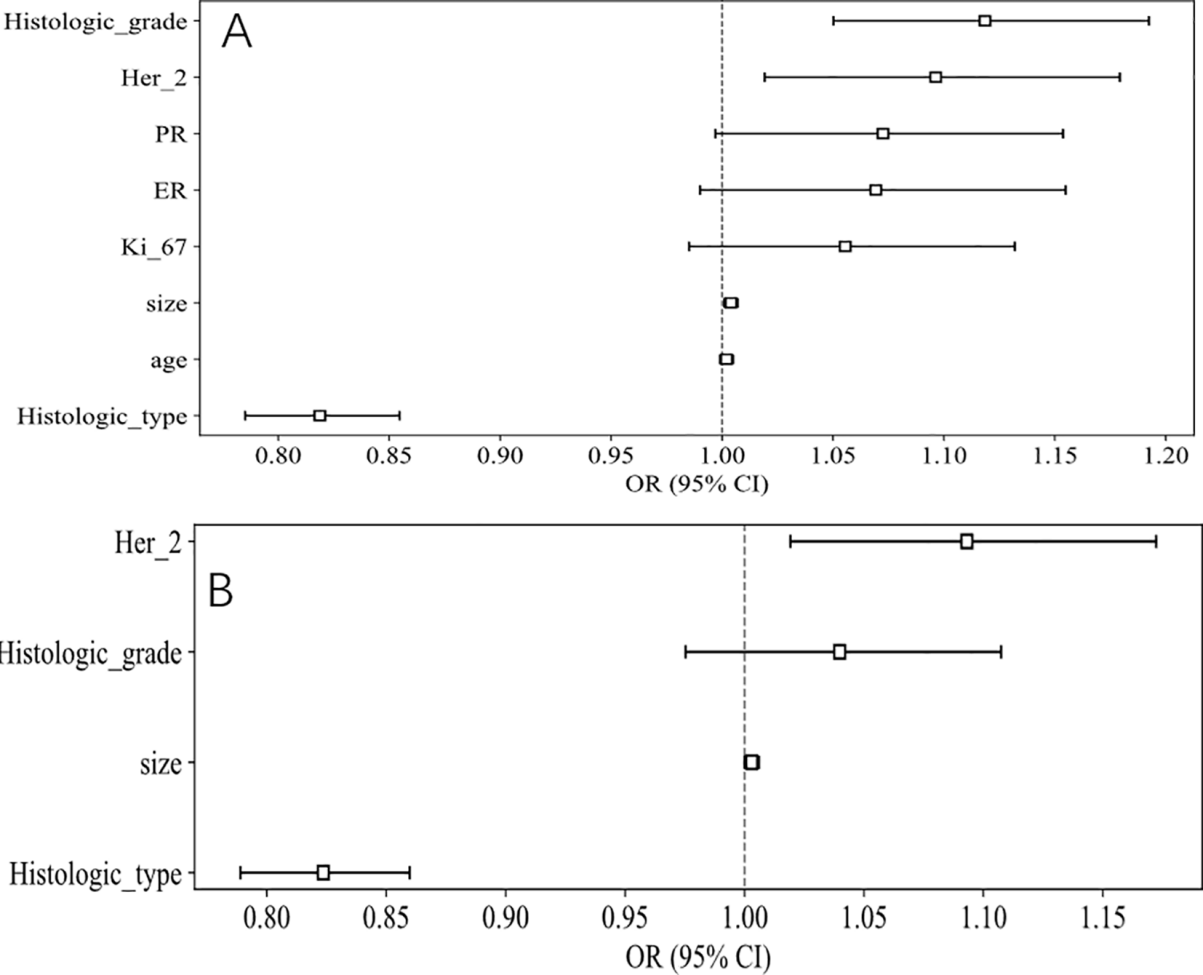
OR of clinical features in univariable and multivariable analysis. **(A)** Univariable analysis. **(B)** Multivariable analysis.

The baseline clinicopathological features of the study cohort are presented in **Table 1**. As shown in **Figure 3** and **Table 2**, notable variations in histologic type and Her2 expression were detected between the cohorts exhibiting positive and negative SLN metastasis across all three datasets (*P* < 0.05). Univariable and multivariable analyses were conducted on all clinical factors, estimating the magnitude of association (OR) and its statistical significance (*p*-value) for each variable. The histologic type (OR = 0.819; 95% CI: 0.785–0.855; *p* < 0.05) and Her2 (OR = 1.097; 95% CI: 1.019–1.179; *p* < 0.05) have been identified as potential predictive factors for SLNM.

**Table 2:** Univariable and Multivariable Analysis of clinical features.

Characteristic	Univariable analyses			p_value	Multivariable analyses			p_value
	OR	OR lower 95%CI	OR upper 95%CI		OR	OR lower 95%CI	OR upper 95%CI	
Histologic_type	0.819	0.785	0.855	<0.05*	0.824	0.789	0.86	<0.05*
age	1.002	0.999	1.005	0.337				
size	1.004	1.001	1.007	<0.05*	1.003	1.0	1.006	0.143
Ki_67	1.056	0.985	1.132	0.198				
ER	1.069	0.990	1.155	0.153				
PR	1.072	0.997	1.154	0.117				
Her_2	1.097	1.019	1.179	<0.05*	1.093	1.019	1.172	<0.05*
Histologic_grade	1.119	1.050	1.192	<0.05*	1.039	0.975	1.107	0.314

ER, estrogen receptor; PR, progesterone receptor; HER-2, human epidermal growth factor receptor 2; OR, odds ratio; 95%CI, 95% confidence interval, *p < 0.05.

### Performance of the automatic ROI segmentation model

**Supplementary Figure 2** demonstrates the relative importance of features in the XGBoost model, highlighting that the DeepLab model’s predictions were the most significant. This correlates with the superior prediction results of DeepLab in individual models. Furthermore, **Table 3** presents a comparative analysis of model performance using various evaluation metrics. The post-fusion model demonstrated reduced segmentation accuracy relative to individual constituent models, yielding Dice scores of 0.889 (training set) and 0.893 (VS). However, an improvement was noted in Dice scores of 0.855 (VS). The model also showed improvement in recall as 0.988 (training set), 0.985 (VS), and 0.956 (TS), respectively. Based on these results, the post-fusion model was selected for constructing image-level features.

**Table 3:** Comparison of different segmentation models with ROI segmentation metrics.

Model Name	Dice	mlOU	Precision	Recall	Cohort
deeplabv3_resnet101	0.929	0.870	0.922	0.941	train
Nested UNet	0.890	0.815	0.853	0.949	train
UNet	0.908	0.838	0.900	0.929	train
Fusion	0.889	0.806	0.815	**0.988**	train
deeplabv3_resnet101	0.924	0.863	0.932	0.924	val
Nested UNet	0.895	0.821	0.863	0.948	val
UNet	0.909	0.841	0.919	0.914	val
Fusion	0.893	0.814	0.826	**0.985**	val
deeplabv3_resnet101	0.842	0.768	0.906	0.851	test
Nested UNet	0.758	0.667	0.902	0.750	test
UNet	0.753	0.672	0.876	0.730	test
Fusion	**0.855**	0.753	0.791	**0.956**	Test

Dice, Dice coefficient or Dice similarity coefficient mlOU, mean Intersection over Union, and the bolded portion is the group with the best indicator.

### Performance of the combined model

#### DL model selection

**Figure 4:**
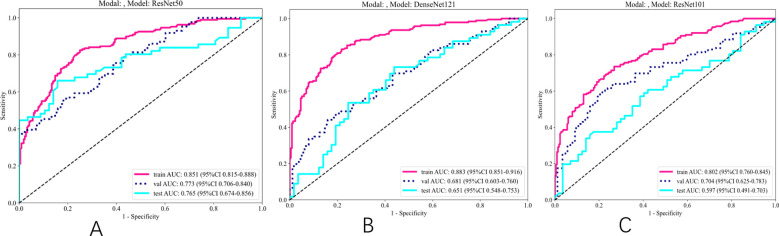
ROC results for deep learning signature of different models. **(A)** ROC of ResNet50. **(B)** ROC of DenseNet121. **(C)** ROC of ResNet101.

As indicated in **Table 4** and **Figure 4**, the ResNet50 model achieved superior performance in predicting SLNM, with accuracy, sensitivity, and specificity in the TS of 74.30%, 64.3%, and 84.2%, respectively. The DenseNet121 model achieved an AUC of 0.651, and the DenseNet101 model recorded an AUC of 0.597. It is worth noting that the ResNet50 model achieved a significantly higher AUC of 0.765 (95%: CI 0.674–0.856) in the TS.

**Table 4:** Metric results for deep learning signature.

ModelName	Accuracy	AUC	95% CI	Sensitivity	Specificity	PPV	NPV	Cohort
resnet50	0.783	**0.851**	0.8148-0 .8880	0.820	0.750	0.742	0.827	train
resnet50	0.684	**0.773**	0.7057-0.8404	0.558	0.807	0.738	0.651	val
resnet50	0.743	**0.765**	0.6739-0.8559	0.643	0.842	0.800	0.706	test
densenet121	0.802	0.883	0.8513-0.9156	0.825	0.782	0.768	0.837	train
densenet121	0.638	0.681	0.6025-0.7602	0.465	0.807	0.702	0.607	val
densenet121	0.637	0.651	0.5485-0.7526	0.714	0.561	0.615	0.667	test
resnetlOl	0.731	0.802	0.7600-0.8446	0.730	0.731	0.704	0.756	train
resnetlOl	0.690	0.704	0.6253-0.7827	0.581	0.795	0.735	0.660	val
resnetlOl	0.602	0.597	0.4914-0 .7028	0.357	0.842	0.690	0.571	test

AUC, area under the curve; 95%CI, 95% confidence interval; PPV, positive predictive values; NPV, negtive predictive values, and the bolded portion is the group with the best indicator.

#### Visual interpretation of the DL model

**Figure 5:**
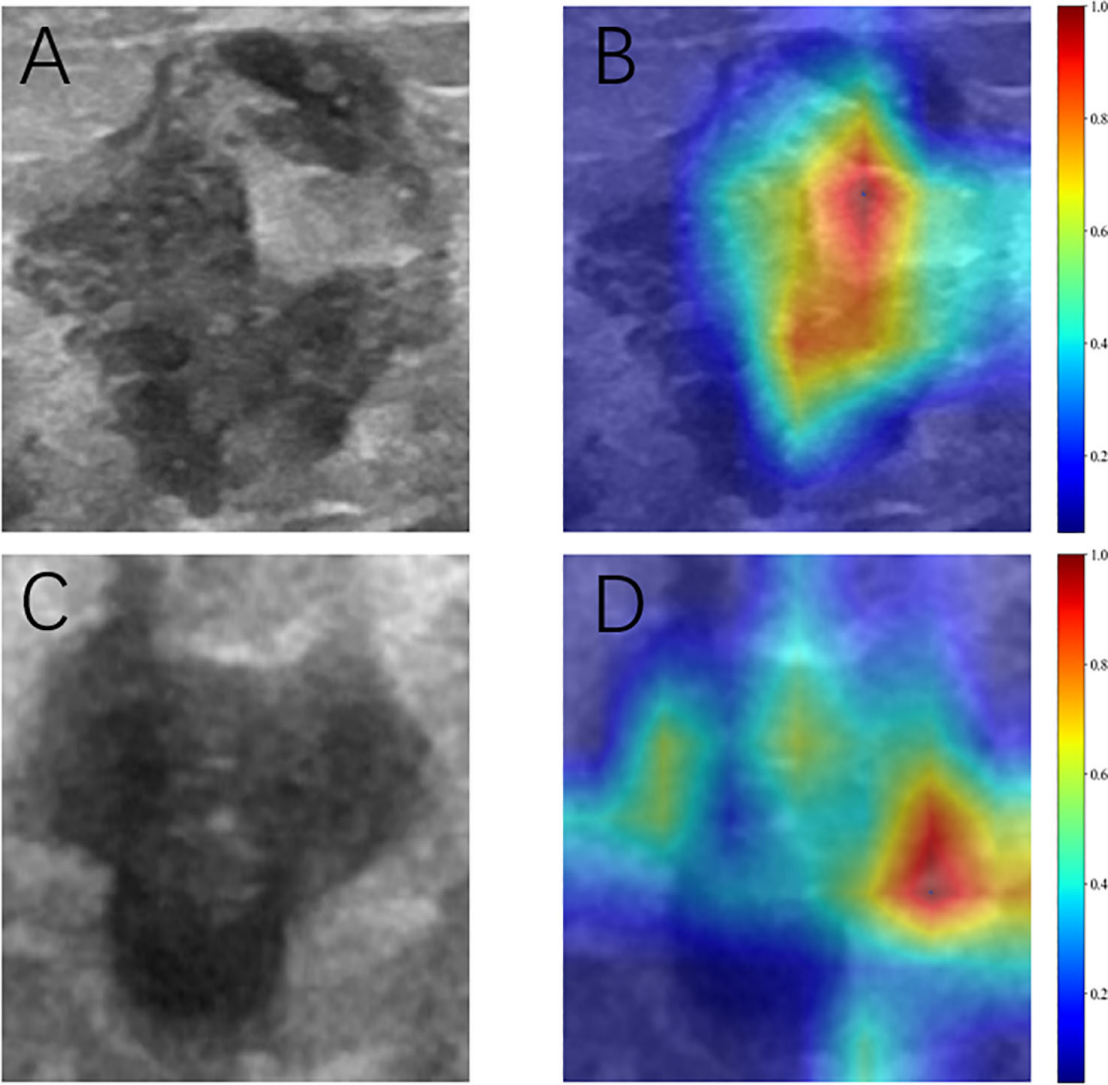
Grad-CAM visualization of two typical samples. **(A, C)** Ultrasonic images. **(B, D)** Corresponding heat maps. The red areas indicate higher contributing, and the blue areas indicate lower contributing for predicting SLN metastasis.

Model interpretability was assessed using gradient-weighted class activation mapping (Grad-CAM) applied to two representative clinical cases identified by the optimal ResNet50 architecture (**Figure 5**). The red areas in the Grad-CAM images highlight the regions that contributed most significantly to the network’s prediction process.

#### Comparison of different models

**Figure 6:**
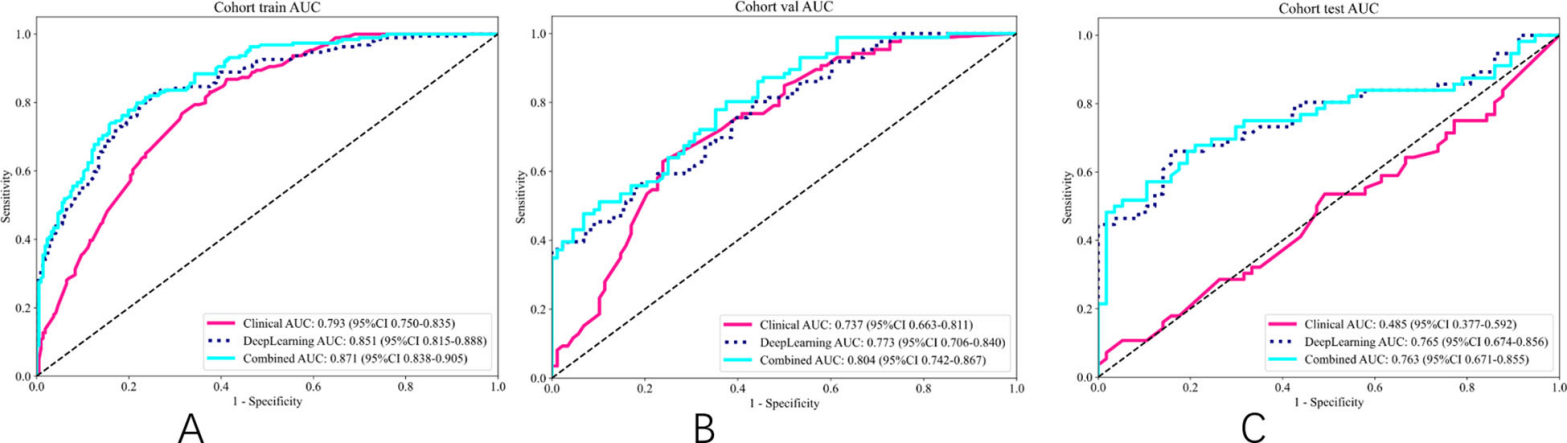
ROC of different signatures on different cohort. **(A)** ROC of training set. **(B)** ROC of validation set. **(C)** ROC of test set.

As shown in **Table 5** and **Figure 6**, the model integrating clinical factors and DL Sig achieved optimal diagnostic accuracy in both the training and validation sets. However, in the TS, the combined model did not demonstrate a significant improvement in AUC, recording a value of 0.763 (95% CI: 0.836–0.855), slightly lower than the DL model’s AUC of 0.765 (95% CI: 0.674–0.856). This suggests that the clinical features did not perform as well as expected on the TS, which is presented in **Supplementary Figures 3**, **4**, leading to a diminished impact of the fusion model. The lack of improvement in the test set could be attributed to the variability in the clinical features.

**Table 5:** Metrics on different signature.

Signature	Accuracy	AUC	95% CI	Sensitivity	Specificity	PPV	NPV	Cohort
Clinical	0.719	0.793	0.7498 - 0.8354	0.825	0.625	0.658	0.804	Train
DeepLearning	0.780	0.851	0.8148 - 0.8880	0.725	0.829	0.787	0.775	Train
Combined	0.790	**0.870**	0.8363 - 0.9033	0.767	0.810	0.780	0.799	Train
Clinical	0.655	0.737	0.6630 - 0.8113	0.767	0.545	0.623	0.706	val
DeepLearning	0.672	0.773	0.7057 - 0.8404	0.744	0.602	0.646	0.707	val
Combined	0.695	**0.804**	0.7417 - 0.8668	0.721	0.670	0.681	0.711	val
Clinical	0.522	0.485	0.3773 - 0.5923	0.536	0.509	0.517	0.527	test
DeepLearning	0.619	0.765	0.6739 - 0.8559	0.821	0.421	0.582	0.706	test
Combined	0.690	**0.763**	0.6707 - 0.8553	0.750	0.632	0.667	0.720	test

AUC, area under the curve; 95%CI, 95% confidence interval; PPV, positive predictive values; NPV, negtive predictive values,and the bolded portion is the group with the best indicator.

#### Comparison with radiologists

The efficacy of the integrated model was assessed in relation to the evaluations provided by four radiologists, as shown in **Supplementary Figure 5**. The findings indicated that the integrated model surpassed the assessments made by clinicians throughout the training, validation, and test sets. Furthermore, the DeLong test (*p* < 0.05) validated that the integrated models significantly outperformed the capabilities of both junior and senior radiologists. The ROC curves along with the DeLong test results for each model are displayed in **Supplementary Figures 6**, **7**.

### Clinical use of the combined model

#### Nomogram construction

**Figure 7:**
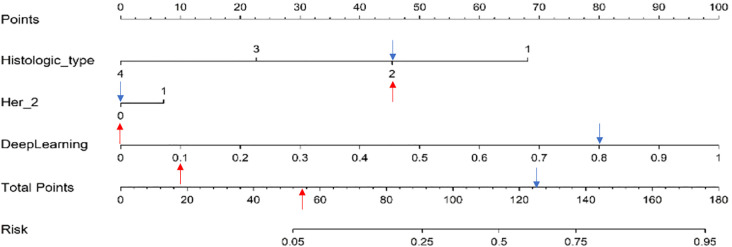
Nomogram construction.

Multivariable regression analysis in the training cohort identified histologic type, Her-2, and DL signatures, respectively, as independent variables of SLN status. These variables were incorporated into the nomogram which is shown in **Figure 7**. Using the nomogram, we first determined each variable’s points. The total score was then computed by summing the corresponding points of all variables, which was subsequently converted to the predicted probability—for example: we have shown a positive SLN metastasis case (blue arrow in **Figure 7**). It was Her2 negative, histologic type II, and with DL value 0.805. The total score was 45 + 0 + 80 = 125. The corresponding prediction was 62.5%. In contrast, another negative SLN metastasis case is indicated by the red arrow in **Figure 7**. It was Her2 negative, histologic type II, and with DL value 0.105. The total score was 45 + 0 + 10 = 55. The corresponding prediction was 7%.

#### Combined model validation

**Figure 8:**
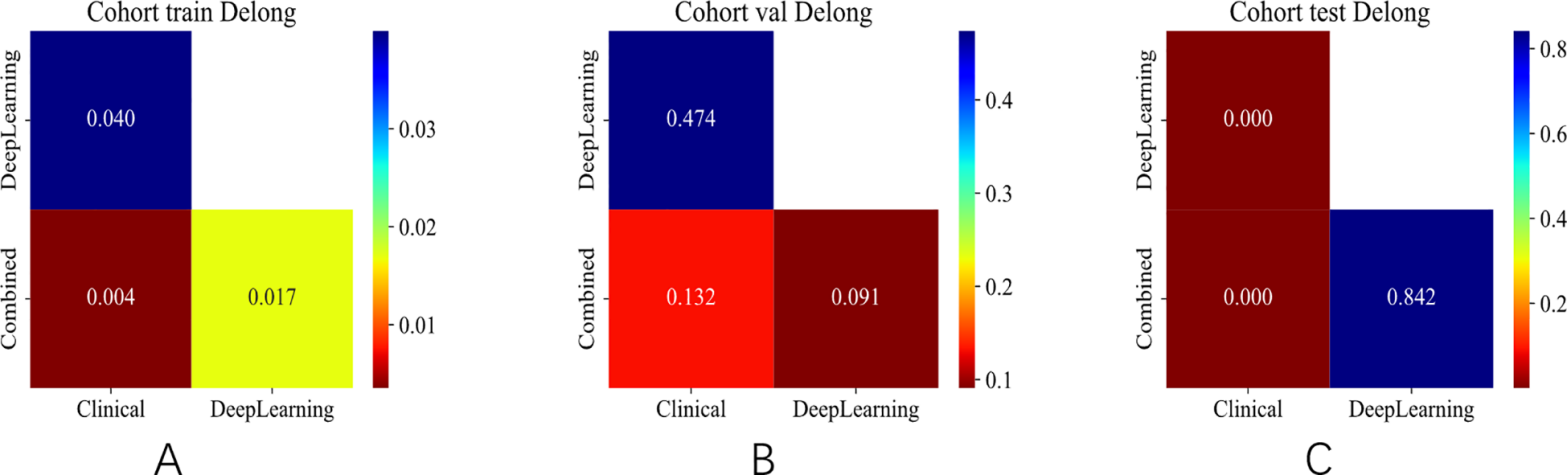
DeLong test of different signatures on different cohorts. **(A)** DeLong test in the training set. **(B)** DeLong test in the validation set. **(C)** DeLong test in the test set.

**Figure 9:**
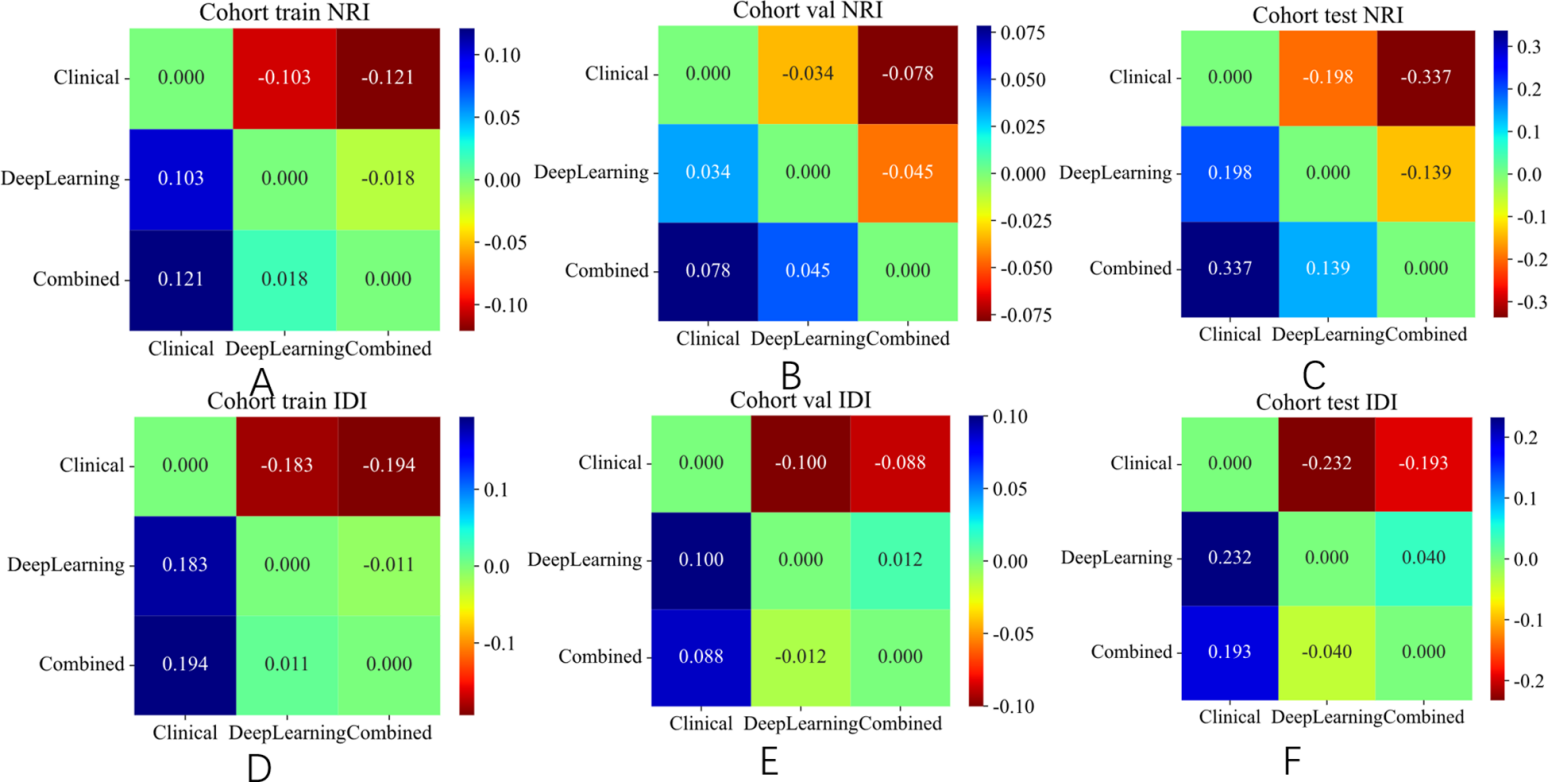
NRI and IDI of different signature on different cohort. **(A)** NRI in the training set. **(B)** NRI in the validation set. **(C)** NRI in the test set. **(D)** IDI in the training set. **(E)** IDI in the validation set. **(F)** IDI in the test set.

**Figure 10:**
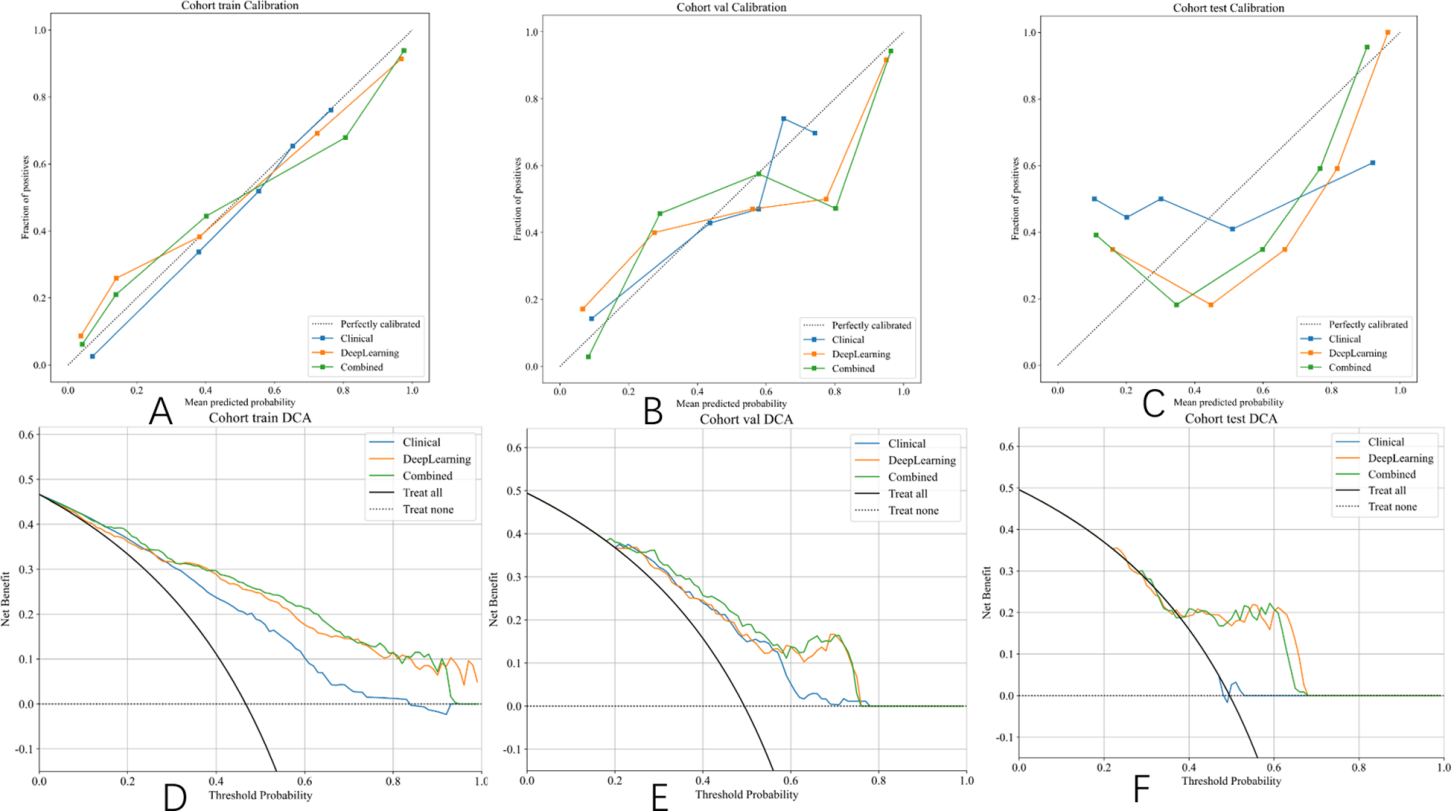
Hosmer–Lemeshow (HL) scores and DCA curve of different signatures on different cohorts. **(A)** Calibration curves in the training set. **(B)** Calibration curves in the validation set. **(C)** Calibration curves in the test set. **(D)** DCA curve in the training set. **(E)** DCA curve in the validation set. **(F)** DCA curve in the test set.

The integrated model significantly outperformed the deep learning model on the TS (DeLong test, Net reclassification improvement (NRI), and Integrated discrimination improvement (IDI), *p* < 0.05 for all) (**Figures 8**, **9**). Both the calibration plots and Hosmer–Lemeshow test confirmed a high degree of concordance between the model’s predictions and the true SLN status (**Supplementary Figure 8**; **Figures 10A–C**). The decision curves illustrated that the integrated model provides greater benefit to patients than either the clinical or DL models alone (**Figures 10D–F**).

## Discussion

SLN status represents a critical prognostic indicator for BC progression and plays a pivotal role in guiding clinical therapeutic decision-making (24). Thus, noninvasive and precise methods for predicting SLN metastatic involvement have become the new research hotspot. Our research successfully presents a deep-learning-based predictive model for SLN metastasis in BC patients. This novel model significantly outperformed senior and junior radiologists. Most notably, to get the full automation of the model, we propose an accurate and robust segmentation model to automatically delineate the ROIs. This novel predicting model was subsequently converted into a nomogram, enabling a quantitative assessment of SLN metastatic risk in BC patients.

US has become an indispensable modality for the morphological evaluation of lymph node characteristics in BC management—including irregular contours, indistinct margins or fatty hilum loss—but its diagnostic efficacy remains constrained to macroscopically detectable abnormalities (25). This inherent limitation results in undetectable micrometastatic deposits within the clinically negative lymph nodes. The precise and effective detection of subclinical nodal metastases is essential for prognostic evaluations, clinical staging, and the refinement of therapeutic approaches. Previous studies (26, 27) demonstrated that decreasing the distance between breast tumors and the skin/nipple complex correlates positively with ALN metastasis incidence. Additionally, architectural distortion (30), lymphatic invasion (31), and calcifications (32) detected on breast US demonstrated a significant predictive value for lymph node metastasis. Traditionally, this requires manual selection of diverse features and their valuation by seasoned radiologists.

Recent studies (33, 34) have highlighted the potential of quantitative US image features derived from primary breast to predict SLN status. Kuo YL et al. (35) developed a validated nomogram predicting non-sentinel lymph node metastasis (NSLNM) risk following positive SLN biopsy, achieving an AUC of 0.738, while Xiu et al. (36) systematically evaluated and compared the predictive performance of machine learning (ML) algorithms versus conventional nomograms for NSLNM detection. Their study found that the XGBoost model achieved superior AUC values compared to conventional nomograms. Additionally, Shahriarirad et al. (37) developed a TabNet-based predictive model for SLN status assessment in BC patients using preoperative clinical variables. Their analysis demonstrated superior predictive performance relative to logistic regression, achieving 75% classification accuracy (versus 70%) and AUC of 0.74 (versus 0.70). Our integrated model, which combined DenseNet50-derived features with clinical factors, demonstrated significant predictive performance, achieving an AUC value of 0.763 and an accuracy level of 69% in the TS. Notably, our model achieved significantly higher predictive accuracy than the assessments by experienced radiologists (AUC, 0.763 vs. 0.708; sensitivity, 75% vs. 70%), demonstrating its potential as an effective tool for preoperative SLN metastasis evaluation.

A key innovation of our study is the development of a fully automated DL model for early-stage ROI segmentation in the image analysis pipeline. In contrast to prior studies dependent on manual segmentation, which are labor-intensive and exhibit substantial inter-observer variability, our approach employs fully automated segmentation, eliminating these limitations. This innovation may optimize clinical workflows by improving radiologists’ efficiency in in BC tumor detection and diagnosis.

Recent advancements in DL have yielded various classical CNN architectures, including FCN, U-Net++, and DeepLabV3+ (29, 30, 38)—for instance, Hu et al. (39) developed an integrated framework combining dilated convolutional networks with phase-based active contour modeling for automated breast lesion segmentation, achieving exceptional performance (Dice coefficient = 88.97%). What is more, Zhao et al. (40) developed MPSegNet for MR-image-based breast tumor segmentation, successfully predicting SLN metastasis with a Dice coefficient of 80% and a sensitivity level of 93.91%. Our study proposed a novel tumor segmentation approach by combined Unet, Unet++, and DeepLabv3, which demonstrated higher Dice of 0.855 and recall of 0.956 in the TS. Our findings were consistent with prior research. This demonstrates that the fusion strategy can compensate for the suboptimal performance of individual models, thereby extending their applicability to a broader range of US imaging scenarios.

Although DL exhibits significant potential, it remains unclear whether incorporating attention mechanisms (e.g., CBAM) to dynamically weight multi-model features would enhance computational efficiency. Furthermore, the extensibility of our approach to other imaging modalities, particularly MRI and PET-CT, warrants systematic investigation. Therefore, subsequent studies should further explore and expand this critical research direction.

Multiple studies have identified clinicopathologic factors that can serve as independent predictors of SLN metastasis. Ding et al. (41) highlighted tumor size, histological grade, and age as significant predictors. Yao et al. (24) demonstrated that high tumor grade and lymphovascular invasion (LVI) positivity independently predict SLN metastasis. The Memorial Sloan Kettering Cancer Center and the MD Anderson Cancer Center nomogram (42) underscored the significant predictive value of patient age and PR status in determining SLN metastasis risk. Shahriarirad et al. (37) emphasized the importance of ER and HER-2 as critical biomarkers in BC classification, which is strongly correlated with SLN metastasis. Our results underscore that histological type and HER-2 exhibit the strongest association with SLN involvement (*p* < 0.05 in univariate analysis), echoing findings from prior research (37, 42). Despite the unique characteristics of BC histological types and the importance of HER-2 as a biomarker, the clinicopathologic model incorporating these factors demonstrated limited predictive power, yielding AUCs of 0.737 and 0.485 in the validation and test sets, respectively. In response to this, we evaluated the integrated model that combined clinical and pathological data, revealing improved predictive accuracy as indicated by AUCs of 0.804 and 0.763 in the validation and test sets, respectively.

Although the current study demonstrated favorable results, several limitations warrant consideration. First, retrospective data collection and sample size constraints may compromise the model’s robustness. Prospective multicenter validation is warranted before clinical deployment. Second, this study did not investigate genomic features associated with SLN metastasis, which could provide valuable insights. Third, patients who underwent neoadjuvant treatment were excluded from this study, potentially limiting the model’s utility to this specific patient group. Fourth, the model’s applicability to patients with non-mass lesions remains untested due to the study’s exclusion criteria. Fifth, despite all US examinations being supervised by experienced physicians, variations in image quality were inevitable. Finally, the study focused solely on image-based automated segmentation DL models to enhance the precision of DL signatures in predicting SLN status. Further research is necessary to determine whether video-based automated segmentation DL models could provide a more reliable preoperative prediction of SLN status.

## Conclusions

In summary, we have developed and validated automated DL segmentation models that significantly enhance the predictive accuracy of DL-derived signatures for SLN status assessment in BC patients. This approach holds potential value in assessing individuals’ risk of SLN metastasis and offering complementary support for guiding personalized therapeutic strategies.

## Data Availability

The original contributions presented in the study are included in the article/**Supplementary Material**. Further inquiries can be directed to the corresponding author.

## Supplementary material

Supplementary Figure 1 Workflow of proposed post-fusion model.

Supplementary Figure 2 XGBoost model’s feature importance.

Supplementary Figure 3 ROC results for clinical signature of different models. **(A)** ROC of LR. **(B)** ROC of random forest. **(C)** ROC of XGBoost.

Supplementary Figure 4 Metric results for clinical signature.

Supplementary Figure 5 Metric results for radiologists and AI on different cohort.

Supplementary Figure 6 ROC and DeLong test results for radiologists and AI on different cohorts. **(A)** ROC of the training set. **(B)** ROC of the validation set.

Supplementary Figure 7 DeLong test results for radiologists and AI on different cohorts. **(A)** DeLong test of training set. **(B)** DeLong test of the validation set. **(C)** DeLong test of the test set.

Supplementary Figure 8 Hosmer–Lemeshow (HL) test statistics for each signature.
