# Supplementary figures and images for: Development of fully automated deep-learning-based approach for prediction of sentinel lymph node metastasis in breast cancer patients using ultrasound imaging
Source: Front Oncol. 2025 Aug 28;15:1592521. doi: 10.3389/fonc.2025.1592521 (PMC12423051; doi:10.3389/fonc.2025.1592521)

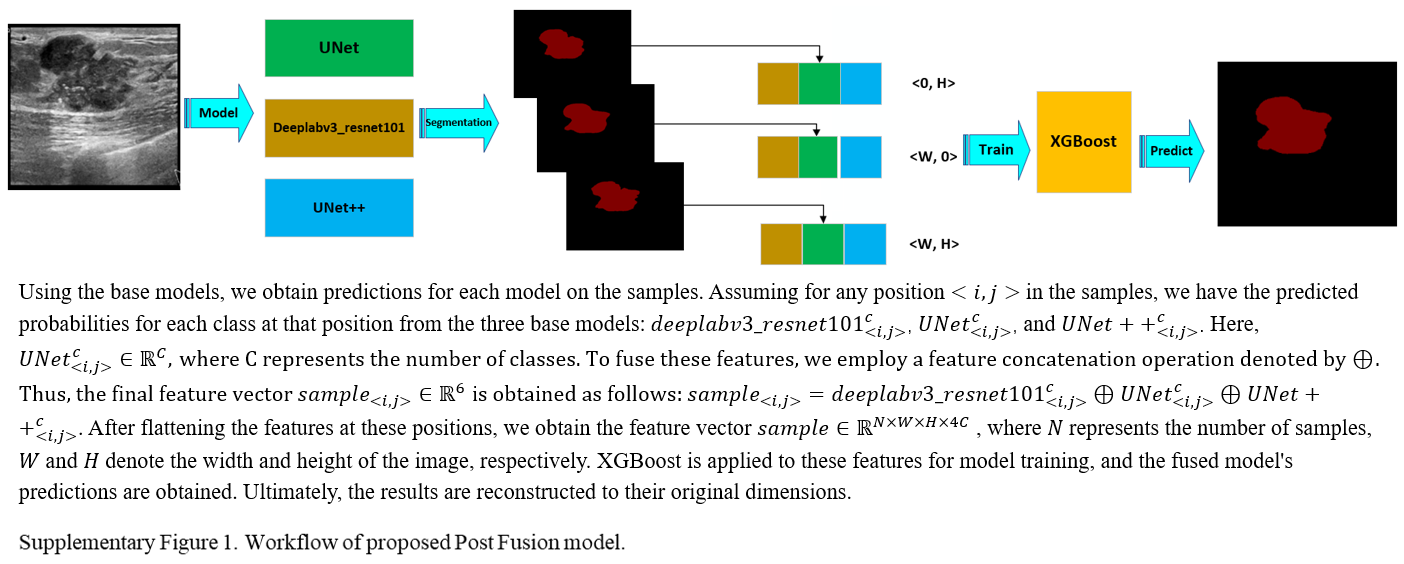

Supplement: Supplementary Figure 1 — Workflow of proposed post-fusion model. [file Image1.png]

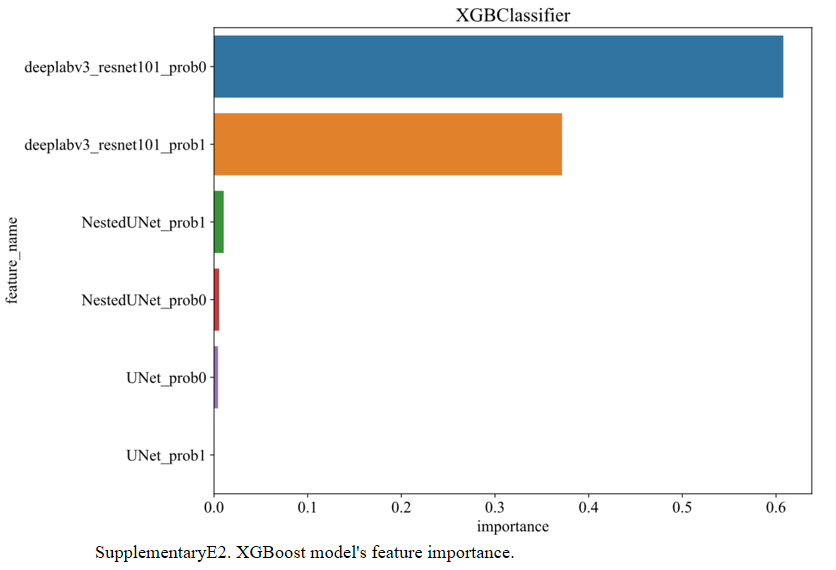

Supplement: Supplementary Figure 2 — XGBoost model’s feature importance. [file Image2.jpeg]

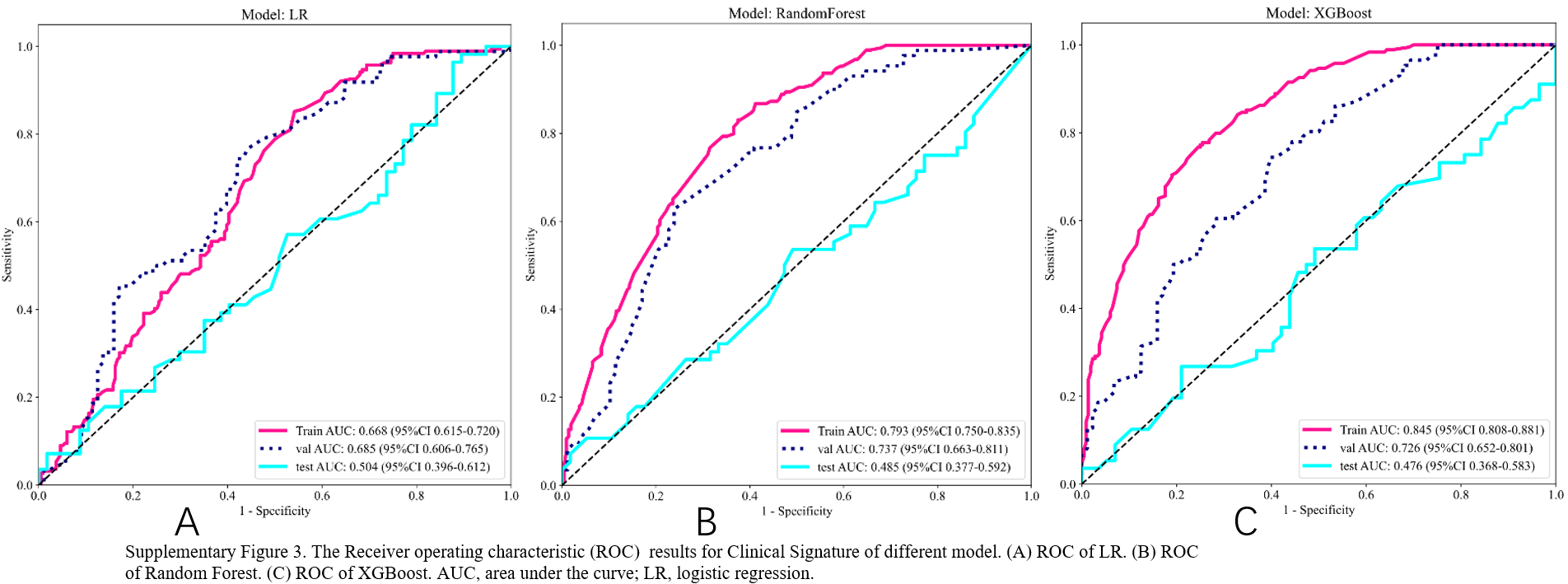

Supplement: Supplementary Figure 3 — ROC results for clinical signature of different models. (A) ROC of LR. (B) ROC of random forest. (C) ROC of XGBoost. [file Image3.png]

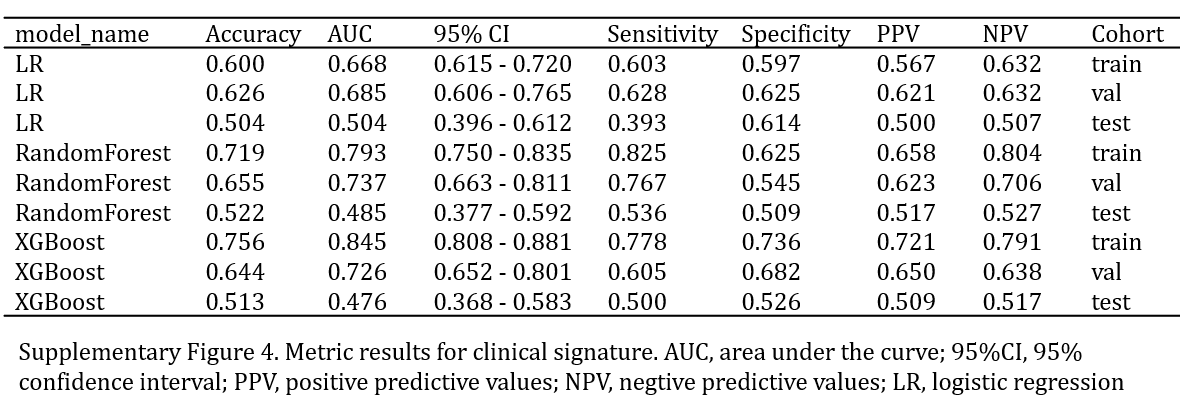

Supplement: Supplementary Figure 4 — Metric results for clinical signature. [file Image4.png]

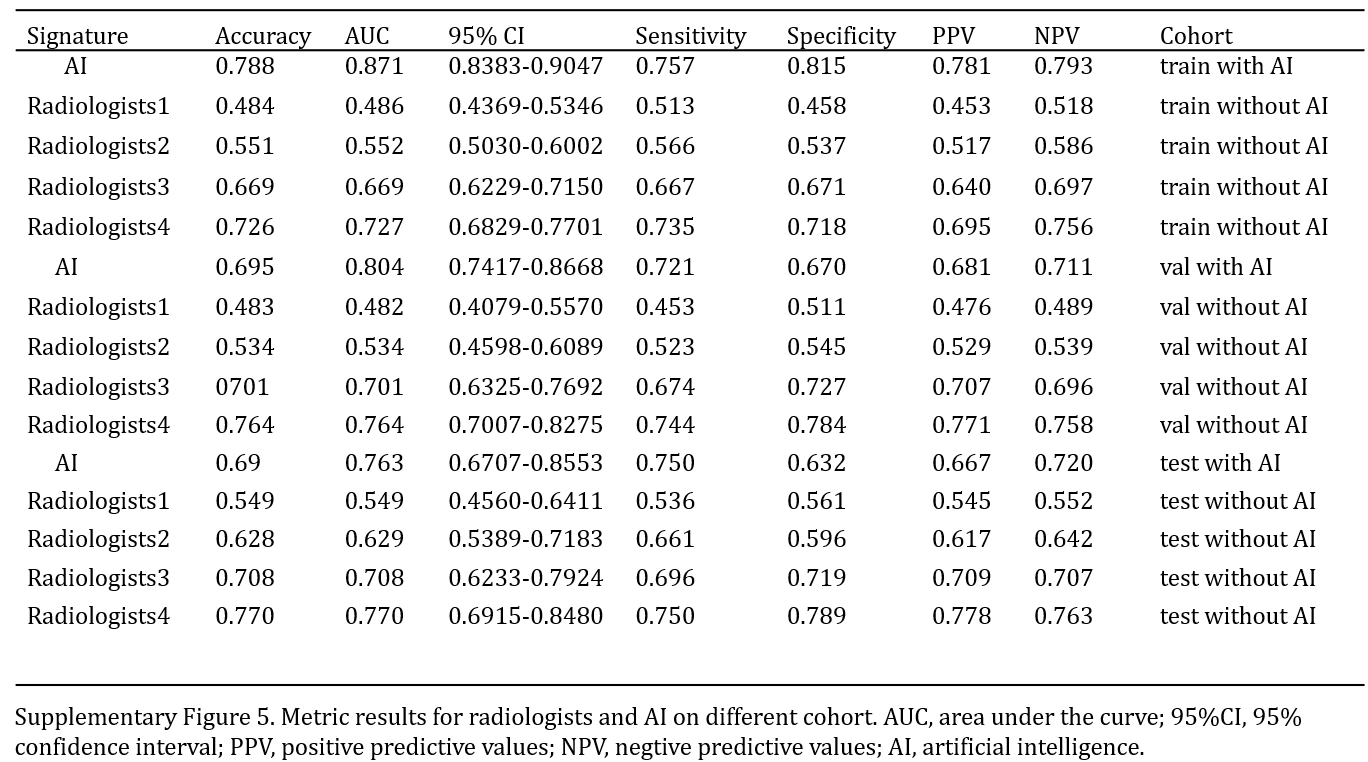

Supplement: Supplementary Figure 5 — Metric results for radiologists and AI on different cohort. [file Image5.png]

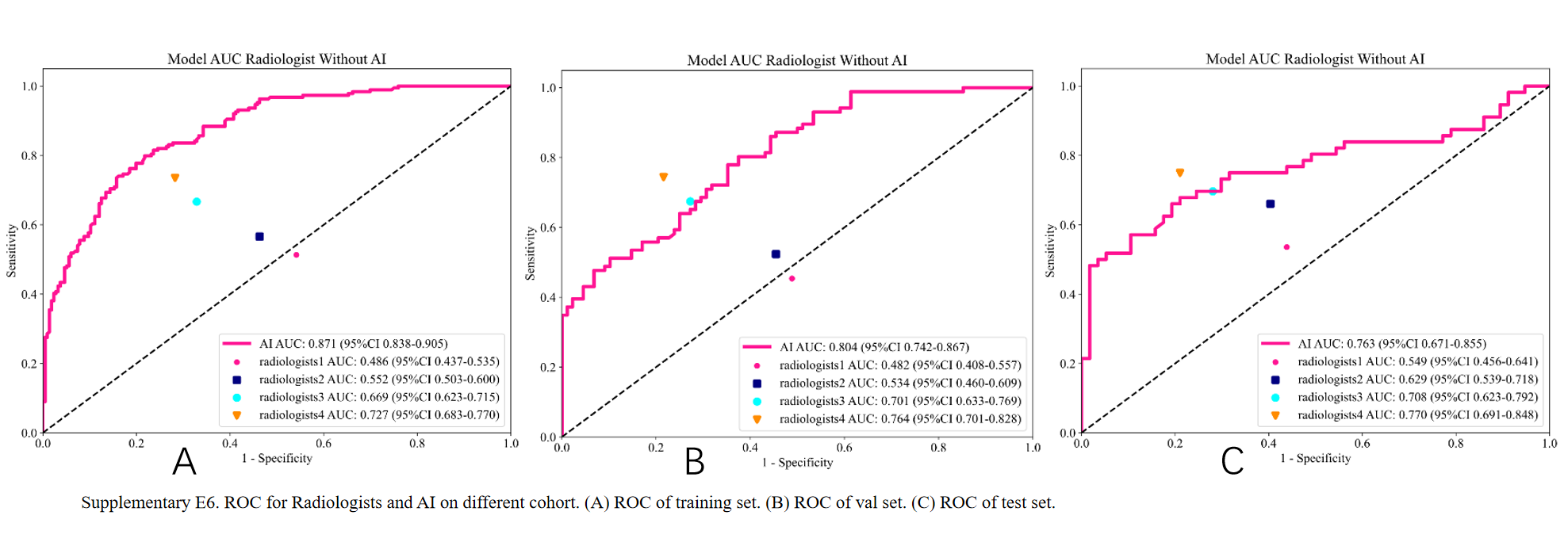

Supplement: Supplementary Figure 6 — ROC and DeLong test results for radiologists and AI on different cohorts. (A) ROC of the training set. (B) ROC of the validation set. [file Image6.jpeg]

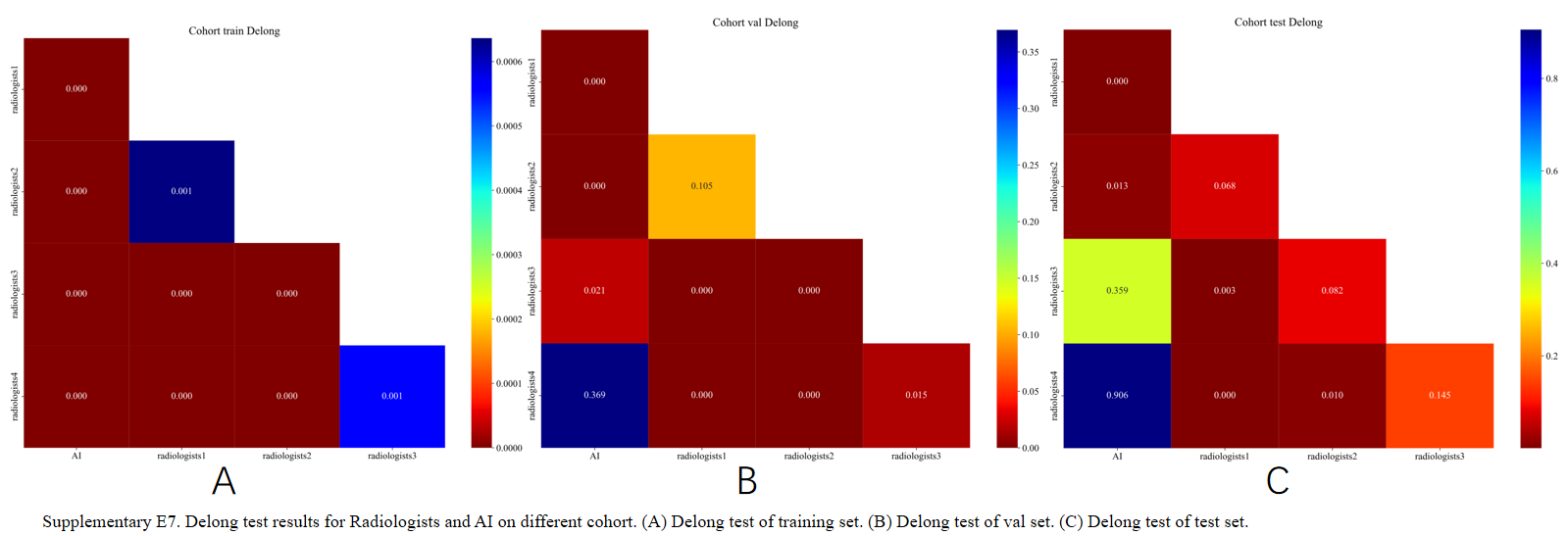

Supplement: Supplementary Figure 7 — DeLong test results for radiologists and AI on different cohorts. (A) DeLong test of training set. (B) DeLong test of the validation set. (C) DeLong test of the test set. [file Image7.jpeg]

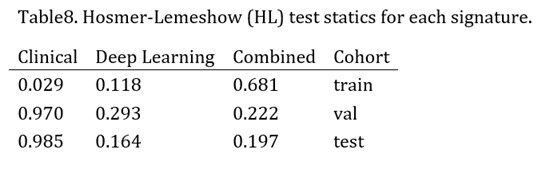

Supplement: Supplementary Figure 8 — Hosmer–Lemeshow (HL) test statistics for each signature. [file Image8.jpeg]

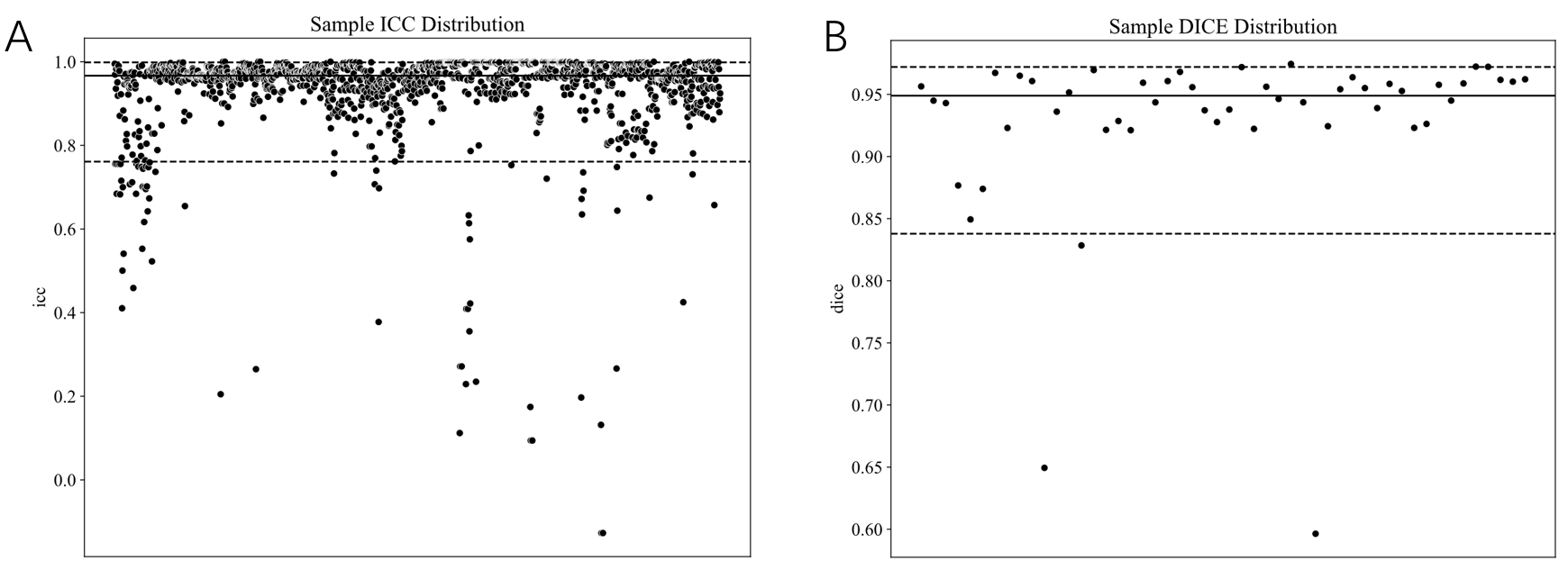

Supplement: Supplementary file 9 [file Image9.png]
